# Supplementary material for: CDK8 inhibitors antagonize HIV-1 reactivation and promote provirus latency in T cells
Source: J Virol. 2023 Sep 28;97(9):e00923-23. doi: 10.1128/jvi.00923-23 (PMC10537590; doi:10.1128/jvi.00923-23)
Supplement: Supplemental material — Fig. S1 to S7. [file jvi.00923-23-s0001.pdf]

Legends to Supplementary Figures

Figure S1. Senexin A and BRD6989 do not affect cell viability at effective concentrations. A, B: Cell viability of JLat10.6 (A) and mHIV-luciferase (B) were determined following incubation with the indicated concentration of Senexin A or BRD6989 for 20 hrs ($n = 2$, mean ± SD). **C, D:** ACH2 (C) and U1 (D) cells were treated for 20 hrs with a DMSO vehicle control (Ve), 10 µM Senexin A, 10 µM BRD6989, 10 nM PMA, or pre-treated for 1 hr with 10 µM Senexin A or10 µM BRD6989 after which 10 nM PMA was added and incubated in combination for 20 hrs ($n = 2$, mean ± SD).

Figure S2. Senexin A and BRD6989 inhibit productive infection in SupT1 cells. A: SupT1 cells were left untreated (Ve) or treated with 10 µM Senexin A or 10 µM BRD6989 and infected with RGH reporter virus. The proportion of productively infected cells was determined at each indicated time point by flow cytometry. Values are normalized to the ratio of productive infections of the vehicle control (Ve) ($n = 2$, mean ± SD). **B:** As in (A), but cellular viability was determined at the indicated time point ($n = 2$, mean ± SD). **C:** Schematic representation of the HIV-GKO dual reporter virus where eGFP is expressed by the 5' LTR and an internal EF1α promoter drives mKO2 expression. **D:** As in (A), but Jurkat cells were infected with HIV-GKO ($n = 2$, mean ± SD).

Figure S3. Knockdown of CDK8 expression by shRNA inhibits reactivation of HIV-1 provirus. A: mHIV-Luciferase Jurkat cells were transduced with vector control (lane 1), or

LKO vector expressing CDK8 specific shRNA (lanes 2-4). Cell lysates were immunoblotted with antibodies against CDK8 (top), or tubulin (bottom) following puromycin selection. **B:** JLat10.6 cells were transduced with vector control (lane 1), or LKO vector expressing CDK8 specific shRNAs (lanes 2-4). Cell lysates were immunoblotted with antibodies against CDK8 (top), or tubulin (bottom) following puromycin selection. **C:** mHIV-luciferase Jurkat cells infected with shRNA as in (A) were left untreated (Ve, DMSO) or treated with 10 nM PMA for 4 hrs when luciferase activity was measured (n = 3, mean ± SD). **D:** JLat10.6 transduced with shRNA as in (C) were left untreated (Ve) or treated with 10 nM PMA for 20 hrs. Following incubation, GFP expression was analyzed by flow cytometry (n = 2, mean ± SD).

Figure S4. Effect of T cell activation on CDK8/19 expression. A: Jurkat mHIV-Luciferase or JLat10.6 cells were left untreated or incubated with 10 nM PMA/ 1 μM Ionomycin for 20 hrs. Cell lysate was extracted and immunoblotted with antibodies against CDK8 (top), CDK19 (middle), and tubulin (bottom). **B, C:** Normalized counts of *CDK8* (A) and *CDK19* mRNA (B) were produced by analysis of a previous RNA-seq experiment that is deposited on NCBI GEO with the accession GSE221851.

Figure S5. ***CDK8* KO and CDK8/19 kinase inhibition differentially impacts HIV-1 expression.** Scatter plots obtained following flow cytometry of RBH infected wildtype or *CDK8* KO Jurkat cells as treated in Fig. 9*A-D*.

Figure S6. Example of gating strategy used for flow cytometry. Threshold forward scatter (FSC) and side scatter (SSC) settings were set so that a homogenous population of living cells was assessed.

Figure S7. Full images of immunoblot scans. Full scans of immunoblot images for production of Figures are shown. Portions of the scans used for production of Figures are indicated (red dashed lines).

Figure S1

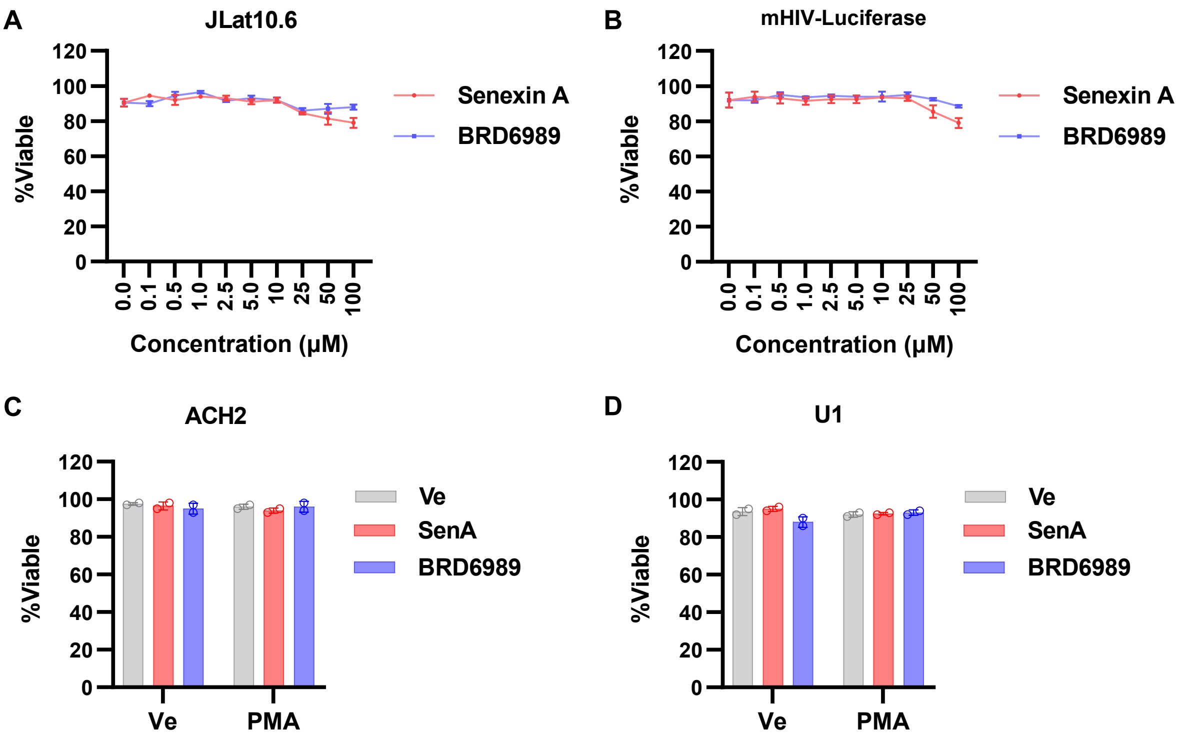

A
JLat10.6
%Viable
120
100
80
60
40
20
0
0.0
0.1
0.5
1.0
2.5
5.0
10
25
50
100
Concentration (µM)
Senexin A
BRD6989
B
mHIV-Luciferase
%Viable
Concentration (µM)
Senexin A
BRD6989
C
ACH2
%Viable
Ve
PMA
Ve
SenA
BRD6989
D
U1
%Viable
Ve
PMA
Ve
SenA
BRD6989

Figure S2

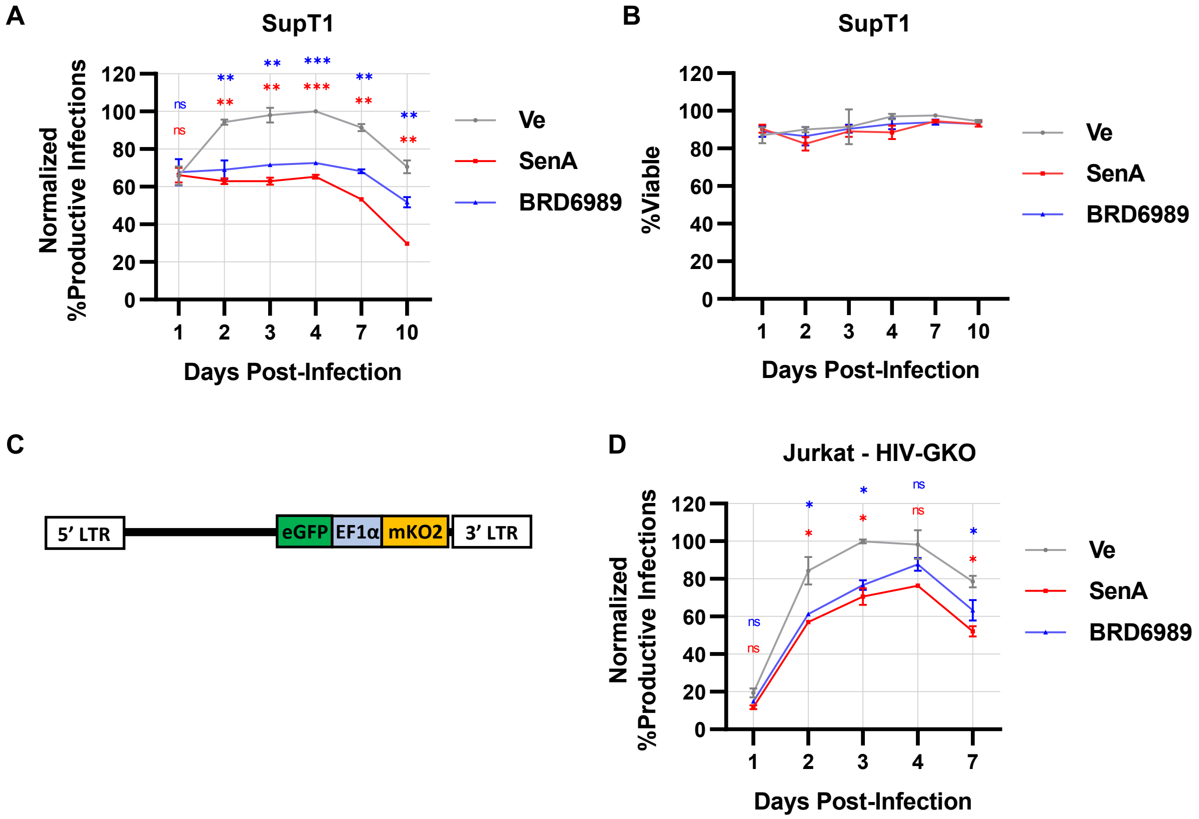
A
SupT1
Normalized %Productive Infections
Days Post-Infection
Ve
SenA
BRD6989
B
SupT1
%Viable
Days Post-Infection
C
5′ LTR
eGFP
EF1α
mKO2
3′ LTR
D
Jurkat - HIV-GKO
Normalized %Productive Infections
Days Post-Infection

Figure S3

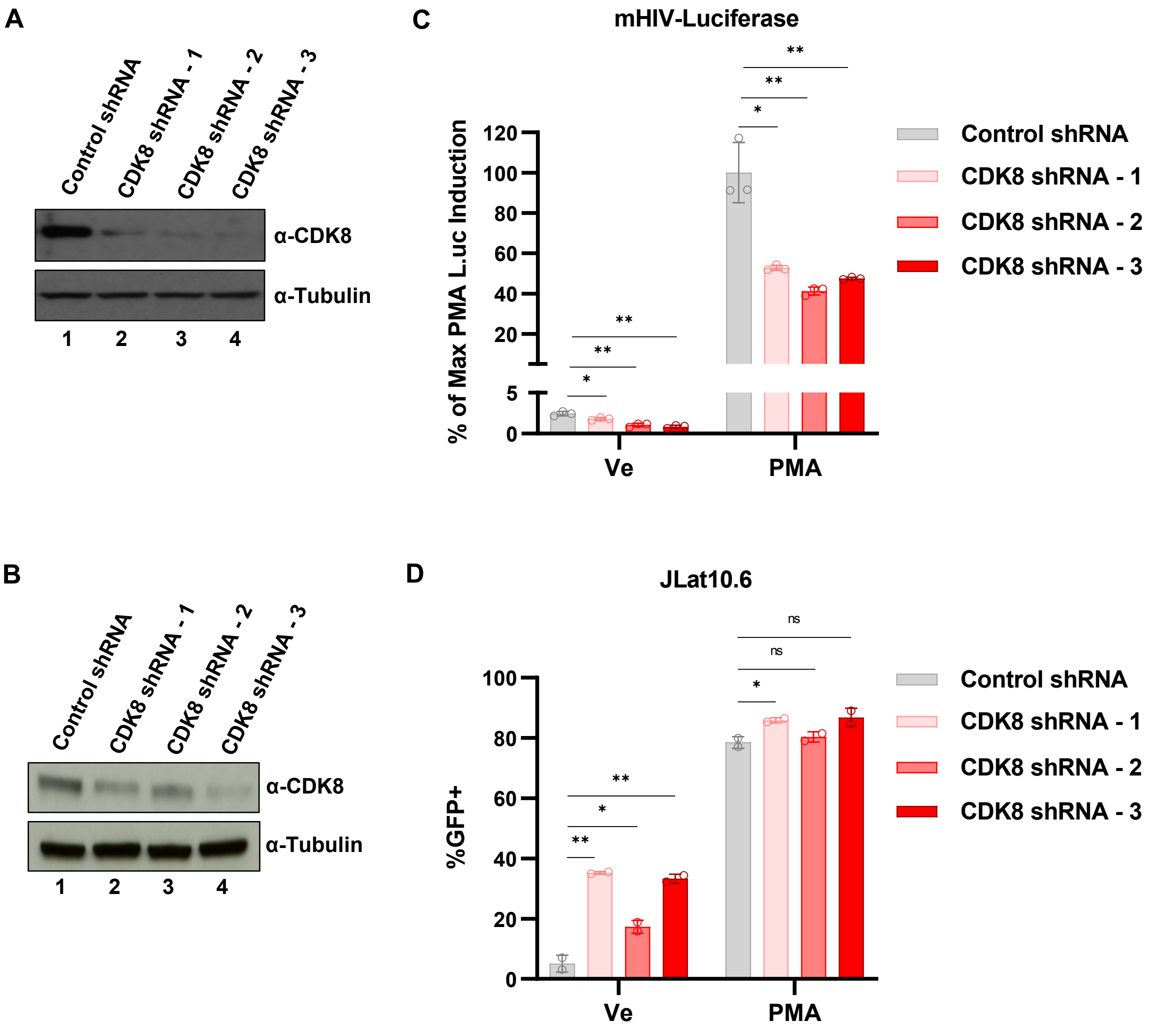

A
Control shRNA
CDK8 shRNA - 1
CDK8 shRNA - 2
CDK8 shRNA - 3
α-CDK8
α-Tubulin
1
2
3
4
C
mHIV-Luciferase
% of Max PMA L.uc Induction
120
100
80
60
40
20
5
0
Ve
PMA
*
**
**
Control shRNA
CDK8 shRNA - 1
CDK8 shRNA - 2
CDK8 shRNA - 3
B
Control shRNA
CDK8 shRNA - 1
CDK8 shRNA - 2
CDK8 shRNA - 3
α-CDK8
α-Tubulin
1
2
3
4
D
JLat10.6
%GFP+
100
80
60
40
20
0
Ve
PMA
**
*
**
*
ns
ns
Control shRNA
CDK8 shRNA - 1
CDK8 shRNA - 2
CDK8 shRNA - 3

Figure S4

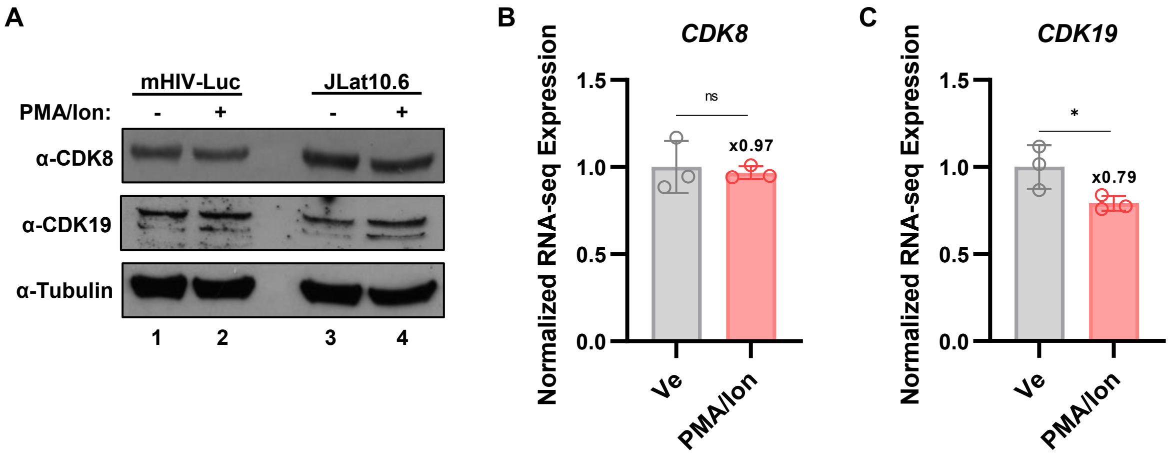

A
mHIV-Luc
JLat10.6
PMA/Ion:
-
+
-
+
α-CDK8
α-CDK19
α-Tubulin
1
2
3
4
B
CDK8
Normalized RNA-seq Expression
1.5
1.0
0.5
0.0
ns
x0.97
Ve
PMA/Ion
C
CDK19
Normalized RNA-seq Expression
1.5
1.0
0.5
0.0
*
x0.79
Ve
PMA/Ion

Figure S5

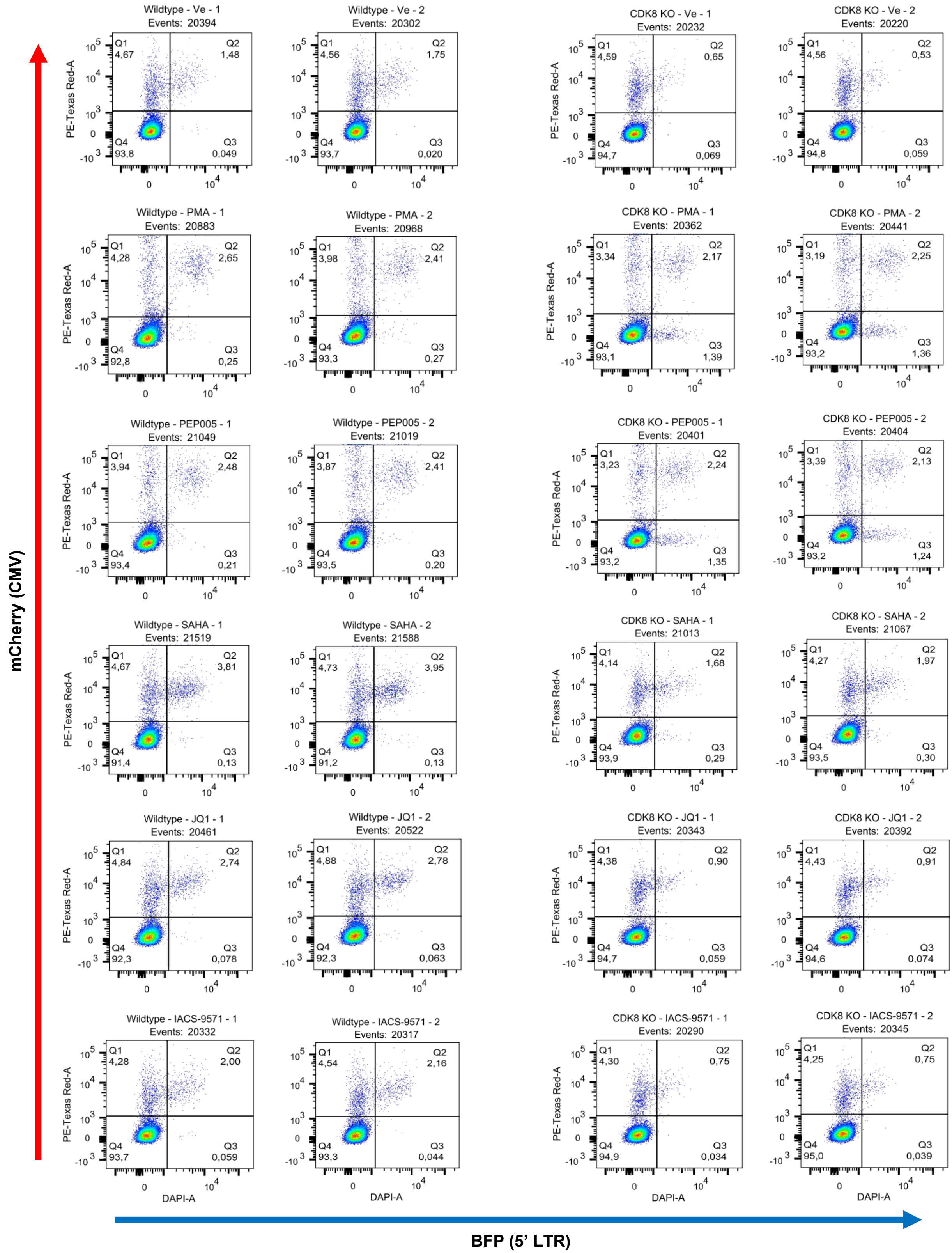

Wildtype - Ve - 1
Events: 20394
Wildtype - Ve - 2
Events: 20302
CDK8 KO - Ve - 1
Events: 20232
CDK8 KO - Ve - 2
Events: 20220
Wildtype - PMA - 1
Events: 20883
Wildtype - PMA - 2
Events: 20968
CDK8 KO - PMA - 1
Events: 20362
CDK8 KO - PMA - 2
Events: 20441
Wildtype - PEP005 - 1
Events: 21049
Wildtype - PEP005 - 2
Events: 21019
CDK8 KO - PEP005 - 1
Events: 20401
CDK8 KO - PEP005 - 2
Events: 20404
Wildtype - SAHA - 1
Events: 21519
Wildtype - SAHA - 2
Events: 21588
CDK8 KO - SAHA - 1
Events: 21013
CDK8 KO - SAHA - 2
Events: 21067
Wildtype - JQ1 - 1
Events: 20461
Wildtype - JQ1 - 2
Events: 20522
CDK8 KO - JQ1 - 1
Events: 20343
CDK8 KO - JQ1 - 2
Events: 20392
Wildtype - IACS-9571 - 1
Events: 20332
Wildtype - IACS-9571 - 2
Events: 20317
CDK8 KO - IACS-9571 - 1
Events: 20290
CDK8 KO - IACS-9571 - 2
Events: 20345
PE-Texas Red-A
DAPI-A
mCherry (CMV)
BFP (5' LTR)

Figure S6

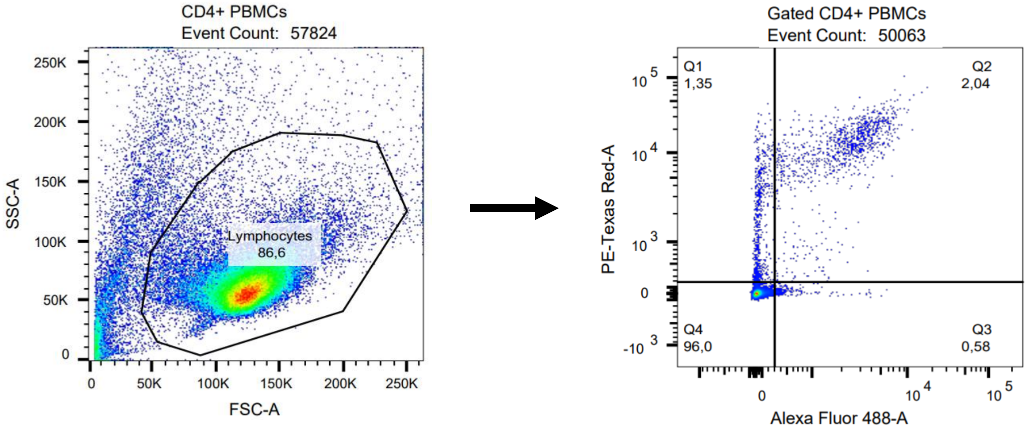
CD4+ PBMCs
Event Count: 57824
SSC-A
FSC-A
Lymphocytes
86,6
Gated CD4+ PBMCs
Event Count: 50063
PE-Texas Red-A
Alexa Fluor 488-A
Q1
1,35
Q2
2,04
Q3
0,58
Q4
96,0

Figure S7

Fig. S3B **α-CDK8**

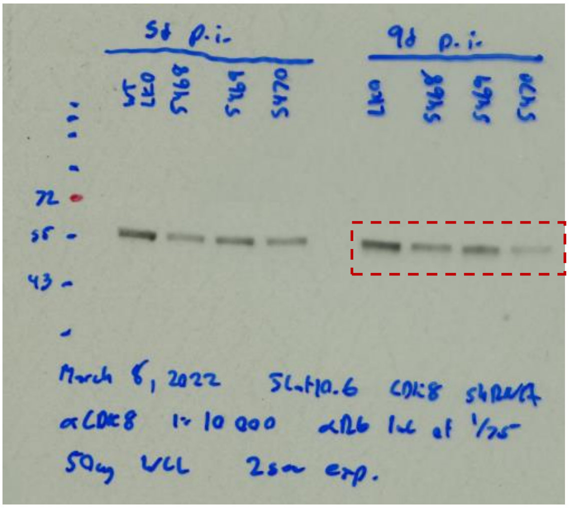

α-Tubulin

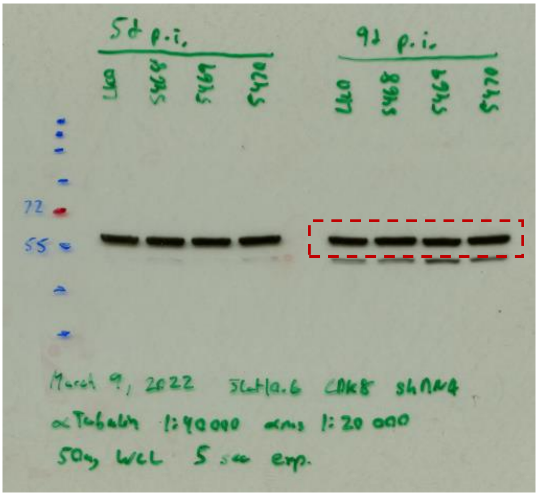

Fig. S4A **α-CDK8**

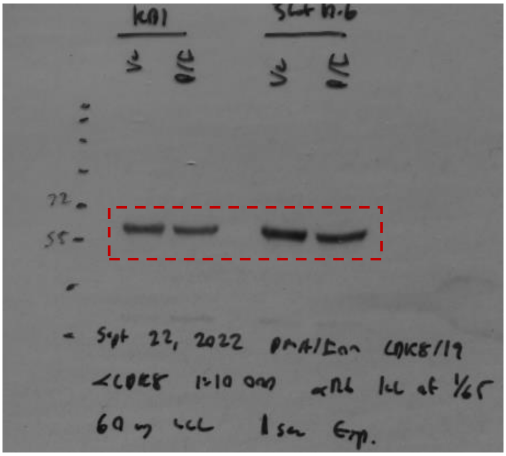

α-CDK19

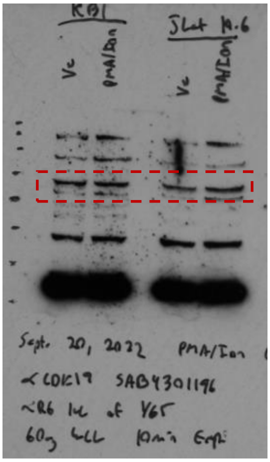

α-Tubulin

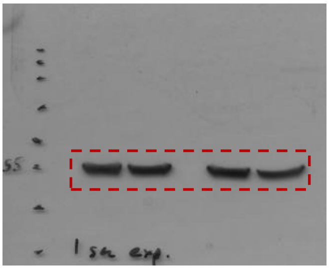

Figure S7

Fig. 8B

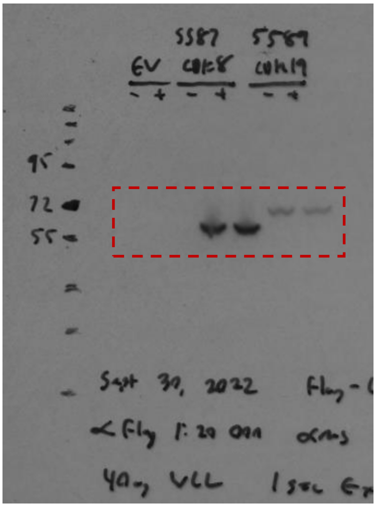

α-Flag

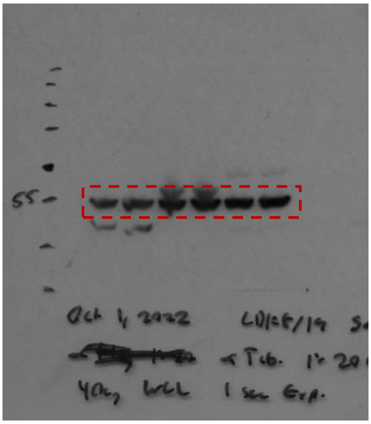

α-Tubulin

Fig. 9A

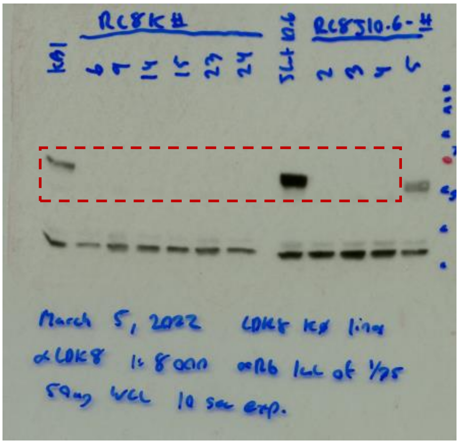

α-CDK8

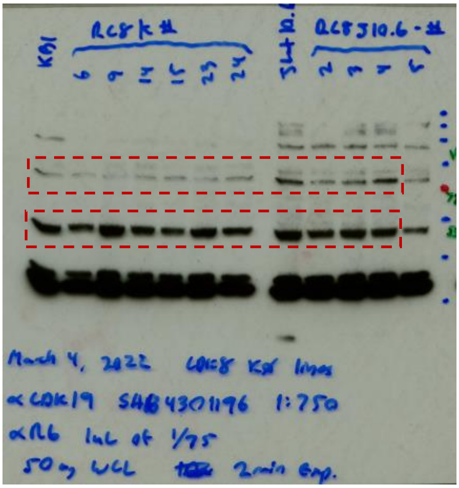

α-CDK19

Fig. S3A

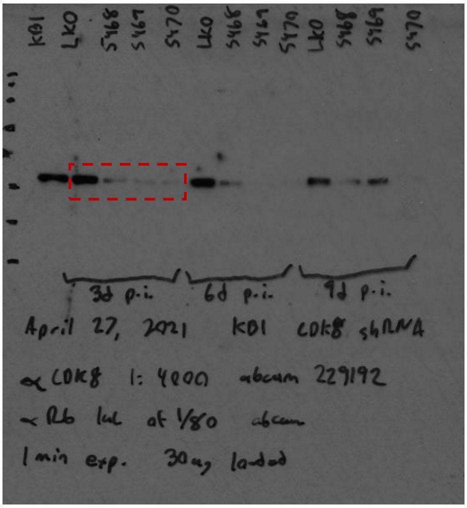

α-CDK8

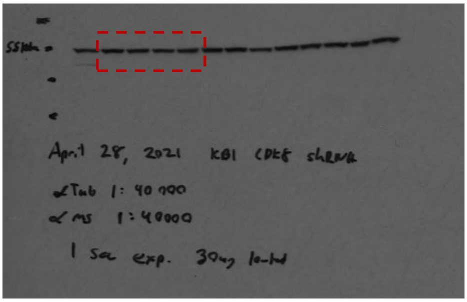

α-Tubulin
